# Phospholipase Dα1 Acts as a Negative Regulator of High Mg^2+^-Induced Leaf Senescence in Arabidopsis

**DOI:** 10.3389/fpls.2021.770794

**Published:** 2021-11-25

**Authors:** Daniela Kocourková, Kristýna Kroumanová, Tereza Podmanická, Michal Daněk, Jan Martinec

**Affiliations:** Institute of Experimental Botany of the Czech Academy of Sciences, Prague, Czechia

**Keywords:** *Arabidopsis thaliana*, magnesium homeostasis, phospholipase D, leaf senescence, starch, proline, abscisic acid, jasmonic acid

## Abstract

Magnesium (Mg^2+^) is a macronutrient involved in essential cellular processes. Its deficiency or excess is a stress factor for plants, seriously affecting their growth and development and therefore, its accurate regulation is essential. Recently, we discovered that phospholipase Dα1 (PLDα1) activity is vital in the stress response to high-magnesium conditions in Arabidopsis roots. This study shows that PLDα1 acts as a negative regulator of high-Mg^2+^-induced leaf senescence in Arabidopsis. The level of phosphatidic acid produced by PLDα1 and the amount of PLDα1 in the leaves increase in plants treated with high Mg^2+^. A knockout mutant of PLDα1 (*pldα1-1*), exhibits premature leaf senescence under high-Mg^2+^ conditions. In *pldα1-1* plants, higher accumulation of abscisic and jasmonic acid (JA) and impaired magnesium, potassium and phosphate homeostasis were observed under high-Mg^2+^ conditions. High Mg^2+^ also led to an increase of starch and proline content in Arabidopsis plants. While the starch content was higher in *pldα1-1* plants, proline content was significantly lower in *pldα1-1* compared with wild type plants. Our results show that PLDα1 is essential for Arabidopsis plants to cope with the pleiotropic effects of high-Mg^2+^ stress and delay the leaf senescence.

## Introduction

Magnesium (Mg^2+^) is a macronutrient involved in essential cellular processes such as photosynthesis, nucleic acid and protein synthesis, energy metabolism, etc. (Guo et al., 2016). Its deficiency or, on the contrary, its excess is a stress factor for plants, seriously affecting plant growth and development. Therefore, accurate regulation of intracellular magnesium level is essential. The knowledge of the mechanisms activated in Mg^2+^ deficiency is relatively good. The mechanisms associated with the regulation of cellular Mg^2+^ under high-Mg^2+^ conditions are less known. High concentrations of Mg^2+^ together with low concentrations of Ca^2+^ occur, for example, in serpentine soils. Recently, high-magnesium water and soils have been considered as an emerging environmental and food security issues (Qadir et al., 2018). For non-adapted plants, high-Mg^2+^ conditions are strongly inhibitory to growth.

Magnesium is absorbed by plants in its ionic form from the soil. Plants growing in soils with high Mg^2+^ can mitigate Mg^2+^ toxicity by limiting internal Mg^2+^ accumulation and/or Mg^2+^ excretion from leaves. Sequestration of additional Mg^2+^ into the vacuole under high Mg^2+^ conditions appears to play a central role in tolerance to high Mg^2+^ (Hermans et al., 2013). The involvement of a network of calcineurin B-like calcium sensor proteins (CBL) CBL2/3, CBL-interacting protein kinases (CIPK) CIPK3/9/23/26, and sucrose non-fermenting-1-related protein kinase2 (SnRK2) SRK2D/E/I in the high-Mg^2+^ response of Arabidopsis has been shown (Mogami et al., 2015; Tang et al., 2015; Chen et al., 2018). Based on the altered sensitivity of the corresponding knock-out mutants to high-Mg^2+^ conditions, several other proteins were identified as participants in the high-Mg^2+^ response. Vacuolar-type H^+^-pyrophosphatase (AVP1; Yang et al., 2018), magnesium transporter 6 (MGT6; Yan et al., 2018), and *mid1*-complementing activity 1, 2 (MCA1/2; Yamanaka et al., 2010) are required for tolerance to high Mg^2+^ because their knock-out mutants were hypersensitive to high-Mg^2+^ conditions. In contrast, knock-out mutants of cation exchanger 1 (CAX1; Cheng et al., 2003; Bradshaw, 2005) and nucleoredoxin 1 (NRX1; Niu et al., 2018) were more resistant to high Mg^2+^. Interestingly, MCA1/2, CAX1, and NRX1 are involved in the regulation of cytosolic Ca^2+^ concentration, suggesting a link between calcium homeostasis and high Mg^2+^ tolerance. In addition, the involvement of ABA signaling in response to high magnesium conditions has been demonstrated. An increase in ABA content and expression of ABA biosynthetic genes was reported under high magnesium conditions (Visscher et al., 2010; Guo et al., 2014). Moreover, the ABA – insensitive mutant *abi1-1* was less sensitive to high magnesium treatment than WT (Guo et al., 2014).

Recently, we discovered that phospholipase Dα1 (PLDα1) activity is vital in the stress response to high-magnesium conditions in Arabidopsis. The T-DNA insertion mutant *pldα1* was hypersensitive to elevated magnesium levels and showed reduced primary root length and fresh weight. PLDα1 activity increases rapidly following high-Mg^2+^ treatment. Moreover, high-Mg^2+^ treatment was shown to disrupt K^+^ homeostasis.

Plant phospholipases D (PLD) cleave common phospholipids such as phosphatidylcholine releasing phosphatidic acid (PA) and free head group. PLDα1, the most abundant PLD member in Arabidopsis, has been reported to play a role in stress responses such as plant-microbe interaction, wounding, freezing, dehydration, and salinity (Wang et al., 2014; Ruelland et al., 2015; Hong et al., 2016). The PA apparently serves as a key signaling molecule in the above responses (Pokotylo et al., 2018).

Leaf senescence is a normal manifestation of plant ageing and represents the final stage of its development. There is also senescence induced by environmental stresses such as drought, cold, heat, low light, and pathogen attack (Zhang and Zhou, 2013; Sade et al., 2018), nutrient deficiencies such as nitrogen (Meng et al., 2016), potassium (Cao et al., 2006; Li et al., 2012; Wang et al., 2012), or magnesium (Tanoi and Kobayashi, 2015). Not only deficiency but also excess of nutrients leads to premature senescence of leaves. Exposure of sunflower plants to elevated K^+^ concentration resulted in premature leaf senescence (Santos, 2001). Ionic imbalance caused by salt stress also causes premature leaf senescence. The regulatory role of ROS (Allu et al., 2014) and transcription factor ANAC092 (Balazadeh et al., 2010) was revealed here. Interestingly, both ROS and ANAC092 are also involved in the regulation of developmental senescence. Thus, there is an overlap between stress-induced senescence and developmental senescence. In addition to ROS and specific transcription factors, the phytohormones jasmonic acid, ABA and cytokinins (CK) also play important roles in senescence processes.

This study shows that high external magnesium concentration triggers leaf senescence in Arabidopsis. Moreover, the knockout mutant of PLDα1 exhibits premature leaf senescence under high-Mg^2+^ conditions compared with WT. Under high-Mg^2+^ conditions, we also observed impaired ion homeostasis of *pldα1*. Furthermore, hormone, starch, and proline accumulation were altered in *pldα1* plants senescing under high Mg^2+^. From these results, we conclude that PLDα1 functions as a negative regulator of high-Mg^2+^ induced leaf senescence.

## Materials and Methods

### Plant Materials

*Arabidopsis thaliana* Col-0 was used in the study. Knockout line *pldα1-1* (SALK_067533) was obtained from the NASC. Complemented lines *pldα1-1*Com1 and *pldα1-1*Com2 were described previously (Kocourková et al., 2020).

### Plant Cultivation

Phenotypic experiments were performed on agar plates and in hydroponics. On vertical agar plates, plants were grown for 10days on ½ MS, 1% agar (Sigma) and then transplanted into either control plates [½ MS (Duchefa), 1% agar] or high-Mg^2+^ plates [½ MS, 15mM MgCl_2_, 1% agar (Sigma)] and grown for another 7days. Plates were kept in a growth chamber at 22°C during the day, 21°C at night, under long day (16h of light) conditions at 100μmolm^−2^ s^−1^ of light. Hydroponic plant cultivation was described in Kocourková et al. (2020). Twenty-four-day-old hydroponically cultivated plants were treated with either ½ Hoagland’s solution (control) or ½ Hoagland’s solution with 15mM MgSO_4_ added. The plants were grown in a growth chamber at 22°C during the day (light intensity of 100μmolm^−2^ s^−1^) and 21°C at night in a 10-h day/14-h night mode.

### PLDα1 Activity

To determine *in vitro* activity, extracts from a mixed leaf sample from the 3rd, 4th, 5th, and 6th oldest true leaves of the plant (=mixed leaf sample) were prepared from hydroponically grown plants treated with 0 or 15mM MgSO_4_ for 1, 2, 3, 4, 7, and 10days. The leaves were frozen in liquid nitrogen. Samples were homogenized and buffer (per 1mg sample 5μl buffer) consisting of 0.4M sucrose, 0.1M MgCl_2_, 0.1M KCl, 50mM HEPES-NaOH pH 7.5, Complete protease inhibitor coctail (Roche) and Pierce Phosphatase Inhibitor Mini Tablets (Thermo Fisher Scientific) was added to the homogenized samples. The samples were centrifuged for 10min at 6,010*g* at 4°C. The supernatant was transferred into a new tube and the samples were centrifuged for 90min at 27,400*g* at 4°C. The supernatant was collected and the protein concentration was measured using a Coomassie Plus Protein Assay (Thermo Scientific).

The enzymatic reaction 100μl contained 15μl of sample (1μg/μl), 50mM MES (pH 6.5, NaOH), 20mM CaCl_2_ and 25μl of substrate solution. The substrate solution contained 4μM fluorescent PC (BODIPY-PC, Invitrogen™ by Thermo Fisher Scientific), 25μM 1,2-dipalmitoyl-*sn*-glycerol-3-phosphocholine (Avanti Polar Lipids), 0.015% sodium deoxycholate and 50mM MES buffer (pH 6.5). The substrate solution was incubated at room temperature for 30min and then sonicated for 10min. The reaction was started by adding the substrate and run for 30min at 25°C with shaking at 500rpm. Lipids were extracted according to Krckova et al. (2018). The lipids were separated first by the mobile phase methanol/chloroform/water/acetic acid (21/15/4/0.8) and after drying by the mobile phase chloroform/methanol/water (26/9/1). The plates were laser-scanned using Sapphire™ Biomolecular Imager (Azzure Biosystems) and evaluated using Azure Spot 2.2 software. The phosphatidic acid standard was prepared using commercial phospholipase D (Sigma Aldrich; Pejchar et al., 2010).

### Western Blot Analysis

Western blot analysis was performed as described previously (Kocourková et al., 2020) with minor changes. Protein extracts were prepared as described above for TLC analysis. Proteins were separated on 10% SDS PAGE and transferred by wet blot overnight on a nitrocellulose membrane. PLDα1 protein was detected with anti-PLDα1/2 antibody (Agrisera) diluted 1: 2000 in 3% low fat milk in TBS-T. Goat anti-rabbit (Bethyl) in 5% low fat milk were used as a secondary antibody. Precision plus protein dual color standard (Biorad) was used and the position of the bands after blot transfer was marked on the membrane with a Western blot marker pen (Abcam). To control protein transfer, the membrane was stained with Novex reversible membrane protein stain (Invitrogen) according to the manufacturer’s instructions.

### Chlorophyll Content

Samples were frozen in liquid nitrogen and homogenized. Chlorophyll was extracted into ethanol. Samples with ethanol were heated to 65°C, left overnight at 4°C and centrifuged (10,000*g*, 10min). The absorbance of the extracts was measured at 649nm and at 665nm and the chlorophyll content was calculated according to Ritchie (2006) and expressed as mg per g fresh weight.

### Gene Transcription Analysis

Gene transcriptions were measured either in whole aboveground parts of plants grown on agar treated with 0 or 15mM MgCl_2_ for 7days or in the mixed leave sample of plants grown hydroponically treated with 0 or 15mM MgSO_4_ for 3days. Measurement of gene expression was done according to Kocourková et al. (2020) with minor changes. Briefly, RNA was isolated using the Spectrum Plant Total RNA Kit (Sigma-Aldrich) and genomic DNA removed using a Turbo DNA-free Kit (Applied Biosystems). Transcription was performed using the Transcriptor First Strand cDNA Synthesis Kit (Roche) with 0.5μg RNA per reaction. Quantitative PCR was performed with a LightCycler 480 SYBR Green I Master Mix (Roche) on a LightCycler 480 System (Roche). The sequences of the primers used are listed in Supplementary Table S1.

### Ion Leakage

Rosettes of plants grown on agar and treated with 0 or 15mM MgCl_2_ for 7days were immersed in deionized water. Electrolyte leakage was measured with a COND 70 portable Conductivity Meter after 1h of incubation at room temperature. The samples were then autoclaved and the total conductivity of the extract was measured. The results were expressed as a proportion of the total conductivity in %.

### Measurement of Nutrient Content

Seedlings were grown for 10days on half-strength MS media, after which they were transferred to agar plates with 0 or 15mM MgCl_2_ for 7days. Plates were kept in a growth chamber at 22°C during the day, 21°C at night, under long day (16h of light) conditions at 100μmolm^−2^ s^−1^. Samples (pooled plants, ~100mg dry weight) were digested with HNO_3_: HCl (6:1, v:v) and P, Mg^2+^, K^+^, and Ca^2+^ content was determined with inductively coupled plasma optical emission spectroscopy (Spectroblue, Spectro, Germany) analysis in the laboratory of Ekolab Žamberk, Czechia.

### Starch Staining

For starch staining the 10-day-old plants grown on agar plates treated for 3days with 0 or 15mM MgCl_2_ and hydroponically grown 24-day-old plants treated for 3days with 0 or 15mM MgSO_4_ were used. Plants were collected at the end of the dark period. Chlorophyll was removed by immersion in 80% hot ethanol. The ethanol was changed until the rosettes were completely discolored. The rosettes were washed with water and then stained for 10min with Lugol solution (Sigma) and washed for 1h in water at room temperature. The plants were then scanned on a Scanner Epson Perfection V800 Photo (Epson).

### Phytohormone Analysis

Phytohormones were analyzed according to Prerostova et al. (2021). Briefly, samples (20–45mg FW leaves) were homogenized and extracted with 100μl 50% acetonitrile solution. The extracts were centrifuged at 4° C and 30,000*g*. The supernatants were applied to SPE Oasis HLB 96-well column plates (10mg/well; Waters, United States) and then eluted with 100μl 50% acetonitrile. The pellets were then re-extracted. Phytohormones in each eluate were separated on Kinetex EVO C18 column (Phenomenex, United States). Hormone analysis was performed with a LC/MS system consisting of UHPLC 1290 Infinity II coupled to 6,495 Triple Quadrupole Mass Spectrometer (Agilent, United States).

### Proline Accumulation Measurement

Proline content was measured in the mixed leaf sample of plants grown hydroponically treated with 0 or 15mM MgSO_4_ for 3, 4, and 7days with ninhydrin method (Bates et al., 1973). The samples were homogenized and proline was extracted into 3% sulfosalicylic acid (SSA, 5μl/mg fresh weight). The samples were centrifuged (5min at maximum speed) and supernatant was collected. The reaction mixture (180μl) consisted of 30μl sample, 96μl glacial acetic acid, 24μl 6M orthophosphoric acid, 30μl 3% SSA and 1.5mg ninhydrin. The reaction was run for 1h at 96°C. Then the samples were cooled on ice and 300μl of toluene was added. The absorbance in the upper phase was measured at 520nm.

## Results

### PLDα1 Activity and Amount of PLDα1 Increase in Leaves of Mg^2+^ Treated Plants

**Figure 1:**
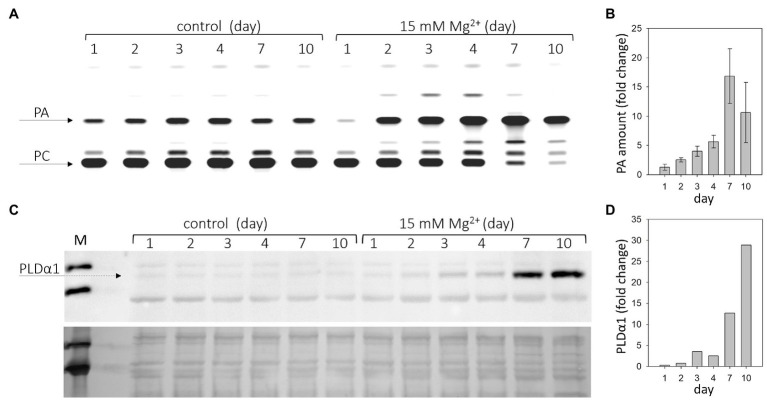
Phospholipase Dα1 (PLDα1) activity and amount increases in response to high Mg^2+^ stress. Twenty-four-day-old hydroponically grown plants were treated with 15mM MgSO_4_ and sampled after 1, 2, 3, 4, 7, and 10days after the treatment. **(A)** Thin layer plate showing phosphatidic acid, product of PLDα1 activity. PLDα1 activity was measured in leave extracts from leaves 3–6 from plants treated with MgSO_4_. **(B)** Relative increase of PA with MgSO_4_ treatment over time. Values represent mean±SE, *n*=3 biological experiments. **(C)** Western blot detection of PLDα1 in protein extracts from leaves. Each lane was run with 15μg of protein, upper panel – western blot, lower panel – loading control – membrane stained with Novex reversible membrane protein stain. **(D)** Quantification of PLDα1 protein. The experiments were repeated three times with similar results. PA, phosphatidic acid; PC, phosphatidylcholine; and M, molecular marker.

We had previously reported that increased Mg^2+^ concentration rapidly induces PLDα1 activity in Arabidopsis roots (Kocourková et al., 2020). Here, we monitored PLDα activity after Mg^2+^ treatment in Arabidopsis leaves. 24-day-old hydroponically grown plants were treated with 15mM MgSO_4_ for 1–10days. Leaves from control and treated plants were harvested, homogenized, and the enzyme activity of PLDα was determined *in vitro*. PLDα activity increased throughout the observation period (1–10days) compared with the control (Figures 1A,B). Higher PLDα1 activity compared with the control was observed after 2days of treatment with 15mM MgSO_4_ (Figures 1A,B) and increased 1.3-fold. The maximum activity was observed on the seventh day, when it increased almost 17-fold.

PLDs cleave common phospholipids such as phosphatidylcholine, releasing PA and the free head group, e.g., choline. PA is also the product of diacylglycerol kinase activity as well as the substrate for PA phosphatase, among other enzymes (Ruelland et al., 2015). Therefore, the PA level does not necessarily correlate with PLD activity. Moreover, there are several isoforms of PLD in Arabidopsis that differ in their biochemical properties (Kolesnikov et al., 2012). To measure PLD activity *in vitro*, we chose the optimal conditions for PLDα activity. PLDα activity was also determined in control and Mg^2+^-treated PLDα1 knockout plants (*pldα1-1*). In *pldα1-1*, no increase in PA level (PLDα activity) was observed under either control or high Mg^2+^ conditions (Supplementary Figure S1). Thus, the activity of the PLDα1 isoform is responsible for the observed increase in PA level. The increase in PLDα1 activity may be due to activation of PLDα1 or a higher level of PLDα1 protein, or both. The amount of PLDα1 in the leaves of control and treated plants was examined by western blot using the anti-PLDα1,2 antibody. The results clearly show that the level of PLDα1 increases after Mg^2+^ treatment (Figures 1C,D). The difference between control and treated plants was detectable after 3days of Mg^2+^ treatment.

Determination of the activity and level of PLDα1 was performed in samples consisting of mature third, fourth, fifth, and sixth leaves. Senescence symptoms were slightly visible in these leaves on the seventh day. However, the same trend, increased activity of PLDα1, was observed in the young leaves (7th–10th), which showed no visible signs of senescence (Supplementary Figure S2). Therefore, we hypothesize that changes in PLDα1 activity and content are not downstream of the manifestation of leaf senescence.

These results show that both PLDα1 activity and PLDα1 levels increase in Arabidopsis leaves after treatment with Mg^2+^.

### High Magnesium Induces Premature Leaf Senescence in pldα1

**Figure 2:**
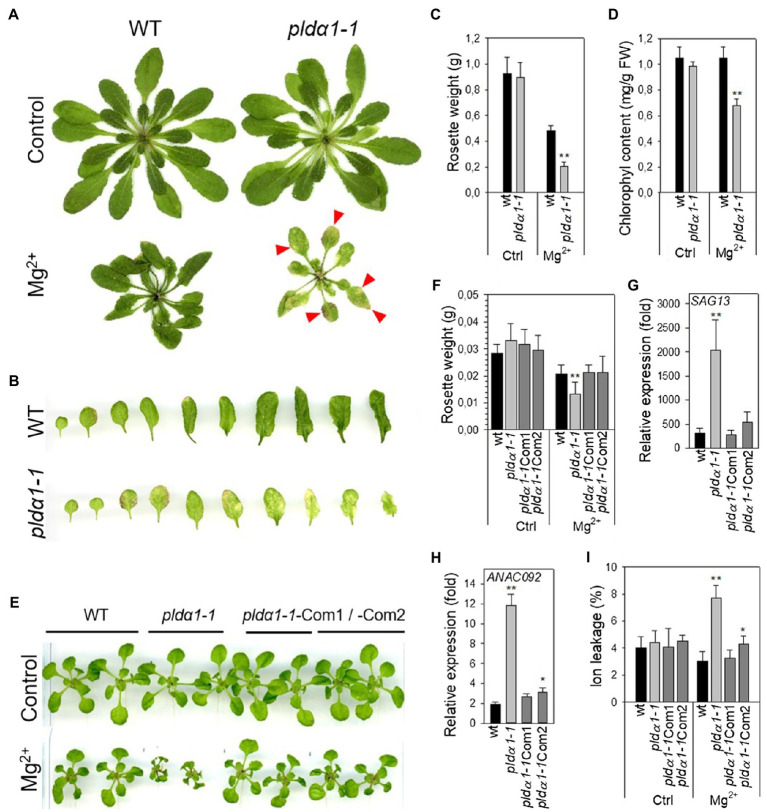
High Mg^2+^ leads to premature senescence in *pldα1-1*. **(A)** Phenotype of WT and *pldα1-1* grown hydroponically on high Mg^2+^, red arrows point to senescent parts of leaves. **(B)** Leaves of plants grown on high Mg^2+^. **(C)** Weight of rosettes. **(D)** Chlorophyll content, (**A–D**) 3-week-old hydroponically grown plants were treated with 15mM MgSO_4_ and grown for another 16days, values represent means±SD, *n*=6. **(E)** Phenotype of WT, *pldα1-1*, *pldα1-1*Com1 and *pldα1-1*Com2 plants grown on agar plates on high Mg^2+^. **(F)** Weight of rosettes. **(G,H)** Transcript level of *SAG13* and *ANAC092* in rosettes. Transcription was normalized to a reference gene *SAND* and the transcription of non-treated plants was set to one. Values represent means±SE, *n*=12. **(I)** Ion leakage, values represent means±SD, *n*=7. **(E–I)** Ten-day-old Arabidopsis seedlings were transferred on agar plates containing 15mM MgCl_2_ and grown for another 7days, Student’s *t* test, asterisk indicate significant difference in comparison with WT ^*^p<0.05; ^**^p<0.01.

We found (Kocourková et al., 2020) that 12-day-old Arabidopsis seedlings of *pldα1* under high-Mg^2+^ conditions had shorter primary and lateral roots and lower fresh weight. Here, we noticed higher yellowing or yellow spots on *pldα1-1* leaves after 15mM MgSO_4_ treatment of 24-day-old plants (Figures 2A,B). We chose 15mM MgSO_4_ for the experiments because no serious adverse effects were observed on the treated plants during the first 4days of treatment. Besides higher yellowing, the fresh weight of *pldα1-1* rosettes was less than half compared with WT, and the chlorophyll content of *pldα1-1* decreased by about 35% compared with WT (Figures 2C,D). This indicates premature leaf senescence of *pldα1-1* plants.

To further characterize the observed phenomenon, we additionally monitored high-Mg^2+^-induced senescence by determining the expression of senescence genes and measuring ion leakage as a marker of membrane damage. To verify that the observed premature senescence was exclusively related to PLDα1, we also included two *pldα1-1*complemented (*pldα1-1*Com1 and Com2) lines (Kocourková et al., 2020). As in the adult plants, the fresh weight of *pldα1-1* rosettes was significantly lower (36%) compared with the WT and complemented lines (Figures 2E,F). Expressions of Senescence-Associated Genes 13 (SAG13, At2g29350) and the transcription factor ANAC092/NAC2/ORE1 (At5g39610) are commonly used as markers of senescence (Balazadeh et al., 2010; Bresson et al., 2018). Expressions of these genes were higher in all Mg^2+^-treated WT, *pldα1-1* and complemented plants in comparison with untreated controls. Hence, high-Mg^2+^ induced transcriptional changes accompanying leaf senescence in all studied genotypes. Moreover, in *pldα1-1* plants, *SAG13* and *ANAC092* expression was notably higher than in WT or complemented lines. Expression of *SAG13* was approximately 2,000-fold higher in *pldα1-1* seedlings treated with high Mg^2+^ than in the untreated control, whereas for WT the increase was only 314-fold higher than in the untreated control (Figure 2G). Similarly, the expression of *ANAC092* was increased 12-fold in *pldα1-1*, whereas it increased only 2-fold in WT (Figure 2H). Membrane damage was estimated by measuring ion leakage. After Mg^2+^ treatment, ion leakage reached 7.7% in *pldα1-1*, while it was only 3% in WT (Figure 2I).

These results demonstrate that high-Mg^2+^ treatment induces premature leaf senescence and that *pldα1-1* plants reveal significantly higher premature leaf senescence in comparison to WT. Premature senescence after high-Mg^2+^ treatment was observed in both 10-day-old seedling and 3-week-old mature plants. Thus, we hypothesize that PLDα1 acts as negative regulator of high-Mg^2+^ induced leaf senescence.

### Levels of Plant Hormones Are Altered in High-Mg^2+^ Conditions

**Figure 3:**
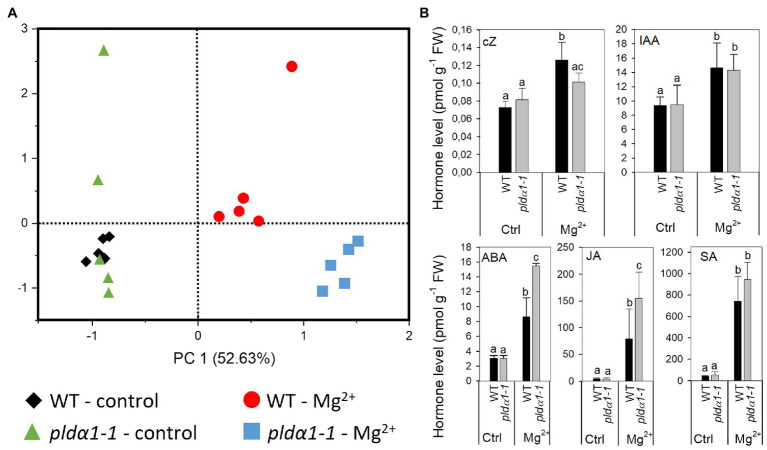
Levels of plant hormone are altered in high-Mg^2+^ conditions. **(A)** PCA analysis. **(B)** Level of cZ, cis-zeatin; IAA, indole-3-acetic acid; ABA, abscisic acid; JA, jasmonic acid; and SA, salicylic acid. Twenty-four-day-old hydroponically grown plants were treated with 15mM MgSO_4_ and grown for another 2days. Leaves 3–6 were used for hormone analysis. Values represent means±SD, *n*=5, letters above the bars indicate significant differences, one-way ANOVA with Tukey’s *post hoc* test, *p*<0.05.

Plant hormones are one of the key components involved in the processes of leaf senescence, influencing all stages, initiation, progression and terminal phase, of leaf senescence (Lim et al., 2007). Additionally, Guo et al. (2014) reported increase level of abscisic acid (ABA) in response of Arabidopsis Landsberg erecta to high-Mg^2+^ conditions. Hence, we measured range of phytohormones in WT and *pldα1-1* in control and high-Mg^2+^ conditions after 2days of high-Mg^2+^ treatment (Supplementary Table S2). Principal component analysis of all measured shoot phytohormones showed a clear separation on the PC1 axis of both control and high-Mg^2+^ conditions and genotypes (WT vs. *pldα1-1*). There was also a separation on the PC2 axis between WT and *pldα1-1* genotype in high-Mg^2+^ conditions (Figure 3A). These results demonstrate robust hormonal response to high-Mg^2+^ conditions in WT as well as involvement of PLDα1 in this hormonal response.

In WT, the highly active cytokinin (CK) trans-zeatin (tZ) and its riboside (tZR) lowered after Mg^2+^ treatment. Also, the content of the precursor trans-zeatin riboside monophosphate (tZRMP) lowered in high Mg^2+^ treated shoots. On the opposite, the levels of the stress-related CKs cis-zeatin (cZ), its riboside (cZR), and phosphate (cZRMP) increased after high Mg^2+^ treatment in WT.

The high-Mg^2+^ treatment up-regulated the production of auxin indole-3-acetic acid (IAA) in WT plants. The level of IAA precursor, indole-3-acetamide (IAM) increased under the same conditions as well. Also, deactivation of production of IAA irreversible amino acid conjugate, IAA-glutamate significantly decreased after high Mg^2+^ treatment in WT.

ABA and its catabolites phaseic acid and 9-hydroxy-abscisic acid (9OH-ABA) elevated about three times in WT shoot of high-Mg^2+^ treated plants in comparison with non-treated plants.

Jasmonic acid (JA) was greatly up-regulated (about 17 times) in high-Mg^2+^ conditions. Similarly, levels of JA precursor, cis-12-oxo-phytodienoic acid (cisOPDA) and JA metabolites, jasmonic acid methyl ester (JA-Me) and dinor-12-oxo-phytodienoic acid (dinorOPDA) significantly increased. Great increase of shoot salicylic acid (SA) level was detected in high-Mg^2+^ treated WT plants as well.

Under control conditions, no significant differences were found between WT and *pldα1-1* in the levels of all hormones measured, except for tZR. Under high-Mg^2+^ conditions, changes of some of the hormones differed between WT and *pldα1-1* (Figure 3B; Supplementary Table S2). Increase of SA was the same in WT and *pldα1-1*. Also, the increase of IAA was the same in WT and *pldα1-1*. However, higher increase was observed in the levels of both IAA precursor, IAM and IAA metabolite oxo-IAA-glucose ester (OxIAA-GE). Increase of JA (but not its precursor or metabolites) was significantly higher in *pldα1-1* in comparison with WT (Figure 3A; Supplementary Table S2). Increase of ABA as well as its catabolites phaseic acid and 9OH-ABA was more pronounced in *pldα1-1* than in WT. Interestingly, increase of cZ detected after Mg^2+^ treatment in WT was not observed in *pldα1-1*.

These results revealed that high-Mg^2+^ condition induce range of hormonal changes in both WT and *pldα1-1* plants. However, changes in ABA and JA levels observed after treatment of plants with high-Mg^2+^ were more pronounced in *pldα1-1* plants. Thus, it suggests that those hormonal changes are, at least partly, under the control of PLD*α*1. It means that the function of PLDα1 in regulation of hormonal changes after high-Mg^2+^ treatment is specific, as the observed difference between WT and *pldα1-1* did not affect all hormones that changed after high-Mg^2+^ treatment of plants but only ABA, JA, and cis-zeatin.

### Ion Homeostasis and Levels of Starch and Proline Are Altered in pldα1-1 Under High-Mg^2+^ Conditions

In our previous work, an imbalance of K^+^ and Mg^2+^ was found in the seedlings of *pldα1-1* treated with high-Mg^2+^. They contained less Mg^2+^ and K^+^ under high-Mg^2+^ conditions (Kocourková et al., 2020). To reveal whether a similar ion imbalance also occurs in high-Mg^2+^-treated shoots, we measured Mg^2+^, Ca^2+^, K^+^, and P in WT and *pldα1-1* shoots under control and high-Mg^2+^ conditions.

**Figure 4:**
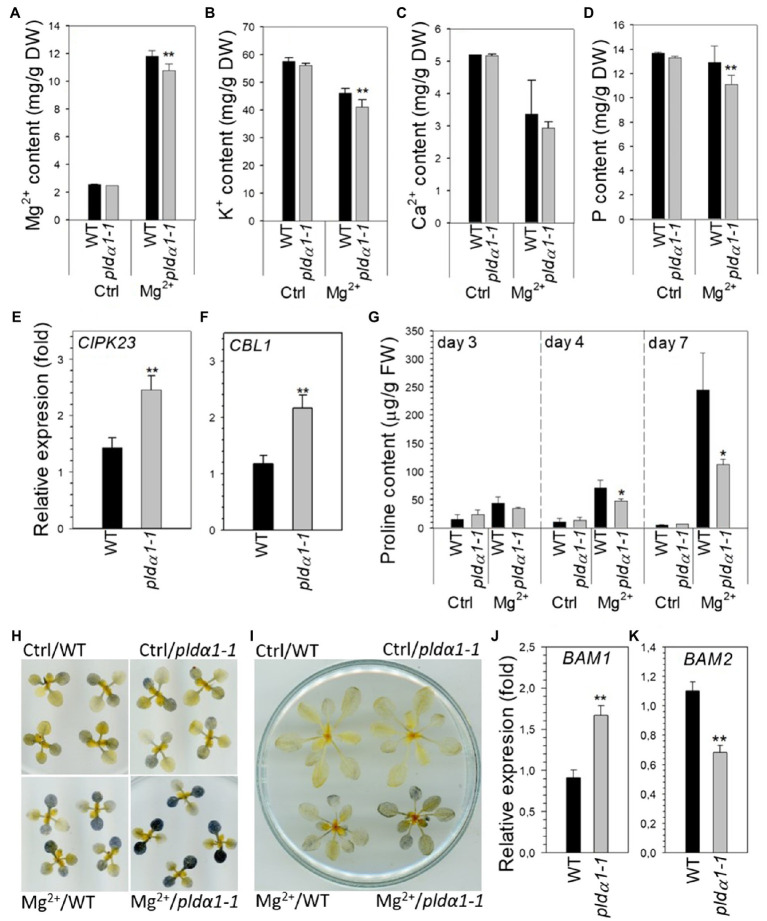
Nutrient, proline and starch content is altered in *pldα1-1* in high-Mg^2+^ conditions. **(A–D)** Magnesium (Mg^2+^), potassium, calcium, and phosphorus content in rosettes of WT and *pldα1-1* plants. Bars represents means±SD, *n*=3 for control, *n*=8 for Mg treatment. **(E,F)** Transcript level of *CIPK23* and *CBL1* in rosettes. Transcription was normalized to a reference gene *SAND* and the transcription of non-treated plants was set to one. Values represent means±SE, *n*=12, (**A–F**) 10-day-old Arabidopsis seedlings were transferred on agar plates containing 15mM MgSO_4_ for 7days. **(G)** Proline content, 24-day-old hydroponically grown plants were treated with 15mM MgSO_4_. Proline content was measured in leaves 3–6. **(H)** Lugol staining of starch in rosettes from control and high Mg^2+^ condition at the end of dark period. Ten-day-old Arabidopsis seedlings were transferred on agar plates containing 15mM MgSO_4_ for 3days. **(I)** Lugol staining of starch in rosettes from control and high Mg^2+^ condition at the end of dark period, 24-day-old hydroponically grown plants were treated with 15mM MgSO_4_ for 3days. **(J,K)** Transcript level of *BAM1* and *BAM2* in leaves 3–6, 24-day-old hydroponically grown plants were treated with 15mM MgSO_4_ for 2days, transcription was normalized to a reference gene *SAND* and the transcription of non-treated plants was set to one. Values represent means±SE, *n*=12. Student’s *t* test, asterisk indicate significant difference in comparison with WT ^*^p<0.05; ^**^p<0.01.

After high-Mg^2+^ treatment (15mM), Mg^2+^ content was increased approximately 5-fold in WT leaves. However, *pldα1-1* showed significantly lower Mg^2+^ content than WT (Figure 4A). Shoot K^+^ content was lower in high-Mg^2+^-treated plants, and *pldα1-1* plants contained even less K^+^ than WT (Figure 4B). Ca^2+^ content was lower in high-Mg^2+^-treated plants, but WT and *pldα1-1* content did not differ (Figure 4C). Furthermore, Niu et al. (2015) showed that the addition of phosphorus to high-Mg^2+^ media resulted in an increase in Arabidopsis root growth and, conversely, the addition of high-Mg^2+^ to low-P media worsened root growth. Based on these results, the authors speculated that the exacerbation of the effects of low P in the presence of high Mg^2+^ was due to the increase in the severity of P deficiency. Therefore, we also measured P content in WT and *pldα1-1* grown on high Mg^2+^ media. Remarkably, the phosphorus content in *pldα1-1* shoots under high-Mg^2+^ conditions was significantly lower than in WT (Figure 4D).

These results were supported by determining the expression of genes known to be associated with ion homeostasis. CBL1 is involved in potassium as well as phosphate homeostasis, whereas CIPK23 is thought to be involved in both magnesium and potassium homeostasis (Ragel et al., 2015; Tang et al., 2015; Gao et al., 2020; Sánchez-Barrena et al., 2020). In WT leaves, the expression of these genes was slightly up-regulated under high-Mg^2+^ conditions (Figures 4E,F). However, in *pldα1-1* leaves treated with high Mg^2+^, *CIPK23* and *CBL1* transcripts were significantly higher than in WT plants.

Proline is well known stress molecule involved mainly in responses to drought and salt stress. Increase of proline content was also reported as response to phosphate starvation in Arabidopsis (Aleksza et al., 2017). As we observed decrease of phosphate content in high-Mg^2+^ treated *pldα1-1* plants we monitor proline level in high-Mg^2+^ treated WT and *pldα1-1* leaves. Proline content substantially increased with increasing time of Mg^2+^ treatment and was tenfold higher in WT plants treated for 7days with high Mg^2+^ than in control plants. Interestingly, shoots of *pldα1-1* contained significantly lower level of proline after Mg^2+^ treatment in comparison with WT (Figure 4G).

It has been reported that that high-Mg^2+^ treatment disturbs starch homeostasis (Guo, 2014) and that both potassium and phosphorus deficiency lead to accumulation of leaf starch (Hermans et al., 2006; Hu et al., 2017). We stained starch with Lugol’s solution in WT and *pldα1-1* seedlings and 24-day-old plants grown under control and high-Mg^2+^ conditions. At the end of dark period, there was clearly a higher starch accumulation in the shoot after Mg^2+^ treatment (Figures 4H,I). Interestingly, higher starch accumulation was observed in *pldα1-1* compared with WT plants. Moreover, shoot expression of β-amylases *BAM1* and *BAM2* was impaired in *pldα1-1* compared with WT under high Mg^2+^ treatment (Figures 4J,K).

These results demonstrate that Mg^2+^, K^+^, and P homeostasis, starch metabolism and proline accumulation are altered in *pldα1-1* shoots of Arabidopsis seedlings grown under high-Mg^2+^ conditions.

## Discussion

In our previous work (Kocourková et al., 2020) we found that *pldα1* plants have shorter roots under high Mg^2+^ conditions compared to WT. We also showed that PLDα1 activity contributes significantly to tolerance to high Mg^2+^. In this work, we focused on the shoots. Our original hypothesis was that PLDα1 activity is important mainly in roots, as they are exposed to high Mg^2+^ conditions and an increase in PLD activity is rapidly induced after high Mg^2+^ treatment (Kocourková et al., 2020). However, we found that PLDα1 activity in the aerial parts of WT Arabidopsis also increased after treatment of the plants with high level of Mg^2+^ ions. Using western blots, we also showed that the amount of PLDα1 increased in shoots treated with high Mg^2+^ and that *pldα1* plants exhibited premature leaf senescence under high Mg^2+^ conditions. Thus, PLDα1 appears to act as a negative regulator of senescence induced by high Mg^2+^.

### Magnesium-Induced Senescence

Leaf senescence is a highly coordinated process. In addition to age-dependent senescence, there is also stress-induced senescence caused by abiotic (drought, salt, high or low temperature, and nutrient imbalance) and biotic stresses (Sade et al., 2018; Guo et al., 2021). Leaf senescence is associated with membrane and chlorophyll degradation. Leaf yellowing and senescence have been reported to be induced by magnesium deficiency (for a review, see Tanoi and Kobayashi, 2015). In addition, a decrease in chlorophyll content was observed by Yan et al. (2018) under conditions of Mg^2+^ imbalance (Mg deficiency and excess). We observed a greater decrease in leaf chlorophyll content and higher ion leakage in *pldα1* plants than in WT. Also, the transcript level of the senescence marker genes *SAG13* (Dhar et al., 2020) and *ANAC092* (Weaver et al., 1998; Miller et al., 1999; John et al., 2001) was significantly higher in *pldα1* plants, although the expression of both genes was also increased in WT plants in which no signs of senescence were yet evident. Since both genes are among the markers of the onset of senescence, it can be concluded that senescence processes are also initiated in WT upon high Mg^2+^ treatment. However, plants with dead PLDα1 tolerate the stress caused by high Mg^2+^ concentrations much worse than WT, leading to apparent premature leaf senescence.

### The High Mg^2+^ Induced Senescence-Associated Hormonal Changes

Hormones play a critical role in regulating both development and stress-induced senescence. Cytokinins, auxin and gibberellic acid (GA) delay leaf senescence, while ABA, salicylic acid (SA), JA, ethylene and strigolactones (SL) promote leaf senescence (Lim et al., 2007; Guo et al., 2021). The overall hormonal changes we observed after high Mg^2+^ treatment of WT plants were in good agreement with the reported hormonal changes during leaf senescence. We found that after 2days of high Mg^2+^ treatment, there was a decrease in active CK, such as *tran*s-zeatin (tZ) and its riboside (tZR) which is in line with gradual decrease in cytokinin content observed during leaf senescence (Singh et al., 1992; Gan and Amasino, 1996). On the other hand, the content of the stress-related CKs *cis*-zeatin (cZ), its riboside (cZR), and phosphate (cZRMP) increased. An increase in cZ during natural senescence was reported in Arabidopsis and tobacco (Gajdošová et al., 2011; Uzelac et al., 2016). The level of cZ differed in WT and *pldα1* plants under high Mg^2+^ conditions. This suggests that the level of cZ is regulated by PLDα1 under high Mg^2+^ conditions. However, the level of cZ in more senescent *pldα1* plants is lower compared with WT. This is a counterintuitive finding, and further studies are required to clarify this phenomenon.

Abscisic acid is a plant hormone whose level increases significantly after abiotic stresses such as drought and salt stress. During leaf senescence, the level of ABA increases, and exogenous application of ABA induces leaf senescence (Lim et al., 2007; Zhang et al., 2012). We also observed a significant increase in ABA level after high Mg^2+^ treatment in WT and *pldα1* plants, while the increase of ABA was higher in *pldα1* plants than in WT plants. This is consistent with the higher senescence of *pldα1* induced by high Mg^2+^ content, which is also consistent with the observations of Guo et al. (2014). The authors found an increase in ABA content after long-term (14days) exposure of Arabidopsis to high Mg^2+^. They also showed that ABA insensitive plants *abi1-1* were less sensitive to high Mg^2+^ treatment. In our experiments, a significant ABA response was observed after only 48h of exposure. Moreover, transcriptome analysis of Arabidopsis roots treated with high Mg^2+^ revealed increased expression of 9-cis-epoxycarotenoid dioxygenase, an enzyme associated with the biosynthesis of ABA, after 45min of high Mg^2+^ treatment (Visscher et al., 2010). All these results suggest that the increase in ABA content and subsequent ABA signaling are involved in the PLDα1-mediated early responses to high Mg^2+^ conditions.

Salicylic acid (SA) and JA are hormones known primarily for their involvement in plant defence mechanisms against pathogens. However, they are also associated with many responses to abiotic stresses (Miura and Tada, 2014; Raza et al., 2020). It has been shown that the level of SA increases progressively during leaf senescence (Breeze et al., 2011; Zhang et al., 2017) and SA plays a direct role in the onset and progression of leaf senescence (Guo et al., 2021). JA content increases during both natural and induced leaf senescence, and external application of JA induces leaf senescence (He et al., 2002; Seltmann et al., 2010). In our experiments, a significant increase in SA and JA was observed in both WT and *pldα1* plants after high Mg^2+^ treatment, and this increase was more pronounced in *pldα1* plants.

JA signaling has been shown to play a role in the biosynthesis of camalexin (Pangesti et al., 2016), a phytoalexin with a described role in the defence response to a variety of pathogens. Its biosynthesis is also induced by some abiotic treatments such as ROS-inducing compound acifluorfen (Zhao et al., 1998) or UV-B irradiation (Mert-Turk et al., 2003). Interestingly, camalexin content increased after high Mg^2+^ treatment in WT and even more (by 4.5-fold) in *pldα1* plants (Supplementary Table S2). It is not clear what role camalexin might play in response to high Mg^2+^ treatment. However, higher camalexin levels in *pldα1* plants might be related to higher JA levels in *pldα1* plants treated with high Mg^2+^. In addition, camalexin biosynthesis is regulated by MPK6 kinase, which has been shown to be a PA binding protein (Yu et al., 2010).

### Ion Imbalance, Starch and Proline Content and Their Role in Senescence

We found changes in ion content in seedlings (Kocourková et al., 2020) and leaves (this work) of plants treated with high Mg^2+^. At WT, the K^+^ content of plants treated with high Mg^2+^ was lower compared to untreated controls. Moreover, K^+^ content under high Mg^2+^ conditions was significantly lower in *pldα1* plants than in WT. Similarly, *pldα1* and WT plants also differed in P content under high-Mg^2+^ conditions; *pldα1* plants had lower P contents than WT. However, the P content of WT did not differ between control and high-Mg^2+^ conditions.

Potassium deficiency has been reported to induce leaf senescence in Arabidopsis and cotton. Interestingly, there is also strong evidence that JA is involved in potassium deficiency – induced leaf senescence (Armengaud et al., 2004; Cao et al., 2006; Li et al., 2012; Hu et al., 2016). We demonstrated an increase in JA level after high Mg^2+^ treatment in both WT and *pldα1*, and that the accumulation of JA in *pldα1* was higher than that of WT. Further experiments are needed to determine whether potassium deficiency, JA accumulation, and leaf senescence are related.

Leaf starch is synthesized during the day and mobilized during the following night to provide a steady supply of carbon and energy. Starch also mediates plant responses to abiotic stresses such as water deficit, high salinity or extreme temperatures. Most studies considered that starch content in leaves decreases in response to abiotic stresses. However, there are also reports that starch accumulation increases in Arabidopsis under stress (Kaplan and Guy, 2004; Skirycz et al., 2009). In our work, we observed increased starch accumulation under high Mg^2+^ conditions in WT plants, and starch accumulation was even higher under these conditions in *pldα1* plants. The opposite effect of high Mg^2+^ was observed by Guo et al. (2014). The authors found lower leaf starch level in Arabidopsis WT under high Mg^2+^ conditions than under control conditions. It is not clear why such different results occurred. One possible explanation could be the use of different ecotypes and experimental conditions. Guo et al. (2014) used an ecotype (Landsberg erecta), a higher Mg^2+^ concentration (32mM) and long-term high Mg^2+^ stress, while we observed starch in leaves on the third day after treating Arabidopsis plants of ecotype Columbia 0 with 15mM Mg^2+^. The relationship between high starch accumulation and leaf senescence has also been described (Schaffer et al., 1991; Oda-Yamamizo et al., 2016; Huang et al., 2018; Xiao et al., 2020). A possible link between PLDα1 and altered starch accumulation could be the PA binding protein glyceraldehyde-3-phosphate dehydrogenase (GAPDH; McLoughlin et al., 2013), as seedlings with genetically reduced GAPDH activity accumulated higher amounts of starch compared to WT (Yang et al., 2015). Moreover, phosphorus deficiency increases leaf sugars and starch content (Cakmak et al., 1994; Hermans et al., 2006) and we found lower phosphorus content in the leaves of *pldα1* plants compared to WT.

We observed proline accumulation in plants exposed to high Mg^2+^ conditions. Proline is a well-known molecule involved in adaptation to stress by, e.g., balancing cellular redox potential, scavenging free radicals and stabilizing subcellular structures (Szabados and Savoure, 2009; Kaur and Asthir, 2015). A relationship between proline metabolism and leaf senescence has been previously noted and discussed (Zhang and Becker, 2015). Proline content significantly increased in detached rice leaves during senescence (Wang et al., 1982). On the other hand, proline catabolism appears to be up-regulated in Arabidopsis during natural leaf senescence (Funck et al., 2010). Moreover, experiments with inhibition of PLD activity by 1-butanol during salt stress showed that PLD appears to be a negative regulator of the delta-1-pyrroline-5-carboxylate synthase 1 gene, which controls proline biosynthesis (Thiery et al., 2004). We have demonstrated that exposure of *pldα1* to high Mg^2+^ resulted in decreased proline accumulation compared to WT. Thus, PLDα1 appears to be a positive regulator of proline synthesis. Why PLDα1-depleted plants have less proline under high-Mg^2+^ conditions is unclear. The difference in proline content between WT and *pldα1* is significant only after prolonged exposure (4days) to high Mg^2+^, and it is therefore possible that this is a side effect of earlier changes caused by high Mg^2+^ rather than a direct regulation of proline metabolism by PLDα1. However, it is possible that proline helps the plants to cope with the stress caused by high Mg^2+^, and the lower proline content of *pldα1* may contribute to the manifestation of senescence in plants with dysfunctional PLDα1. Interestingly, significant differences in proline content were observed between WT and phosphoenolpyruvate carboxylase 3 (PEPC3) knockout in Arabidopsis under control and salt stress conditions and PEPC3 was identified as the PA-binding protein (Testerink et al., 2004).

### Mechanism of PLDα1 Involvement in Leaf Senescence

PLDα1 has been described to be involved in a variety of biological processes. PLDα1 knockout or antisense-suppressed plants show alterations in water loss, reactive oxygen species production (ROS), response to ABA and stomatal movement (Zhang et al., 2004, 2009; Mishra et al., 2006), salt stress (Bargmann et al., 2009; Yu et al., 2010, 2015), freezing sensitivity (Rajashekar et al., 2006) and seed aging (Devaiah et al., 2007). The involvement of PLDα1 in senescence has also been documented. In 1997, Fan et al. (1997) observed that treatment of detached leaves with ABA and ethylene led to accelerated senescence and increased level of PLDα mRNA, protein and activity. Using the PLDα antisense construct, they then prepared plants with reduced PLDα1 expression. Suppression of PLDα had no effect on natural plant growth and development. Even in the absence of ABA and ethylene, the detached leaves of the PLDα-deficient and WT plants showed similar rate of senescence.

However, the senescence rate of detached leaves of transgenic plants treated with ABA or ethylene was slower than that of detached leaves from WT. Later, Jia et al. (2013) showed that the application of n-butanol, an inhibitor of PLD, and N-acylethanolamine (NAE) 12∶0, a specific inhibitor of PLDα, delayed ABA-promoted senescence to different extents. These data suggest that suppression of PLDα blocks membrane lipid degradation, which ultimately delays ABA-promoted senescence. Thus, PLDα1 appears to be important mediator that play a positive role in phytohormone-promoted senescence in detached leaves. However, in this work, we showed that PLDα1 likely serves as a negative regulator of senescence. We observed that PLDα1 activity and PLDα1 abundance increase during senescence triggered by high Mg^2+^ content. An increase in PLDα1 expression has also been described during age-related leaf senescence (Xiao et al., 2010). However, in our case, the increase in PLDα1 activity and PLDα1 abundance is probably not related to the increased membrane degradation described above, because *pldα1-1* plants exhibited significantly higher senescence compared with WT. Therefore, another regulatory mechanism by which PLDα1 is involved in the regulation of senescence induced by high Mg^2+^ levels must play a role.

In general, there are two molecular ways by which PLDα1 may regulate other events. The first is linked with PLDα1 activity which leads to the production of the second messenger phosphatidic acid and free head group, and the second is protein–protein interaction. In the case of PLDα1, both scenarios have been documented. A combination of both mechanisms is also possible and has been described in the case of PLDα1 involvement in ABA responses (see below). PLD-derived phosphatidic acid is produced in response to various biotic and abiotic stresses such as plant defence, wounding, salt, drought, cold, and heat stress (Yao and Xue, 2018). In salt stress PA produced by activated PLDα1 binds to ABI1, a protein phosphatase 2C, a negative regulator of the ABA response and inhibits its function (Zhang et al., 2004). Since ABA functions as a positive regulator of leaf senescence, PLDα1 could also play the role of a positive regulator of leaf senescence. However, in our study, this is not the case because PLDα1 is a negative regulator of leaf senescence induced by high Mg^2+^ content.

A number of PA-binding proteins have been found (Yao and Xue, 2018). CTR1 (CONSTITUTIVE TRIPLE RESPONSE1) is another example of PA binding protein (Testerink et al., 2004, 2007). CTR1 is a Ser/Thr protein kinase that functions as a negative regulator of ethylene signaling. Loss of CTR1 function has been shown to promote the senescence process upon dark treatment, suggesting that CTR1 plays a role as a negative regulator of leaf senescence (Li et al., 2017). We did not measure ethylene levels in our experimental setup. However, ethylene is known to be an endogenous modulator of senescence, including leaf senescence.

PLDα1 protein interaction, the second possible PLDα1 regulatory mechanism, is also involved in the regulation of ABA responses. PLDα1 interacts with components of heterotrimeric G protein signaling, GPA1 (Gα) and Gβ proteins (Zhao and Wang, 2004; Gookin and Assmann, 2014). The interaction of PLDα1 with GPA1 stimulates the GTPase activity of GPA1 (Zhao and Wang, 2004). PLDα1 also interacts with RGS1 protein (regulator of G protein signaling). RGS1 likely inhibits the GAP activity of PLDα1 (Choudhury and Pandey, 2016). To further impact the specificity of this pathway, PA, the product of PLDα1 activity, binds to RGS1 and inhibits its GAP activity. Interestingly, GPA1-, Gβ- as well as RGS1 knock-out plants showed altered salt stress-induced senescence (Colaneri et al., 2014).

In summary, high Mg^2+^ induces leaf senescence and many of the physiological changes associated with leaf senescence induced by high Mg^2+^ are under the control of PLDα1. Subsequent studies should elucidate the precise molecular mechanism of this PLDα1 control.

## Data Availability Statement

The original contributions presented in the study are included in the article/Supplementary Material, further inquiries can be directed to the corresponding author.

## Author Contributions

DK and JM designed the study and wrote the manuscript. DK, KK, TP, and MD performed the experiments. All authors contributed to the article and approved the submitted version.

## Funding

This research was supported by the Czech Science Foundation (grant No. 17-00522S) and the Ministry of Education, Youth and Sports of the Czechia (European Regional Development Fund-Project “Centre for Experimental Plant Biology” no. CZ.02.1.01/0.0/0.0/16_019/0000738).

## Conflict of Interest

The authors declare that the research was conducted in the absence of any commercial or financial relationships that could be construed as a potential conflict of interest.

## Publisher’s Note

All claims expressed in this article are solely those of the authors and do not necessarily represent those of their affiliated organizations, or those of the publisher, the editors and the reviewers. Any product that may be evaluated in this article, or claim that may be made by its manufacturer, is not guaranteed or endorsed by the publisher.
