# Supplementary material for: Phospholipase Dα1 Acts as a Negative Regulator of High Mg2+-Induced Leaf Senescence in Arabidopsis
Source: Front Plant Sci. 2021 Nov 25;12:770794. doi: 10.3389/fpls.2021.770794 (PMC8656112; doi:10.3389/fpls.2021.770794)
Supplement: Supplementary file 2 [file Data_Sheet_1.pdf]

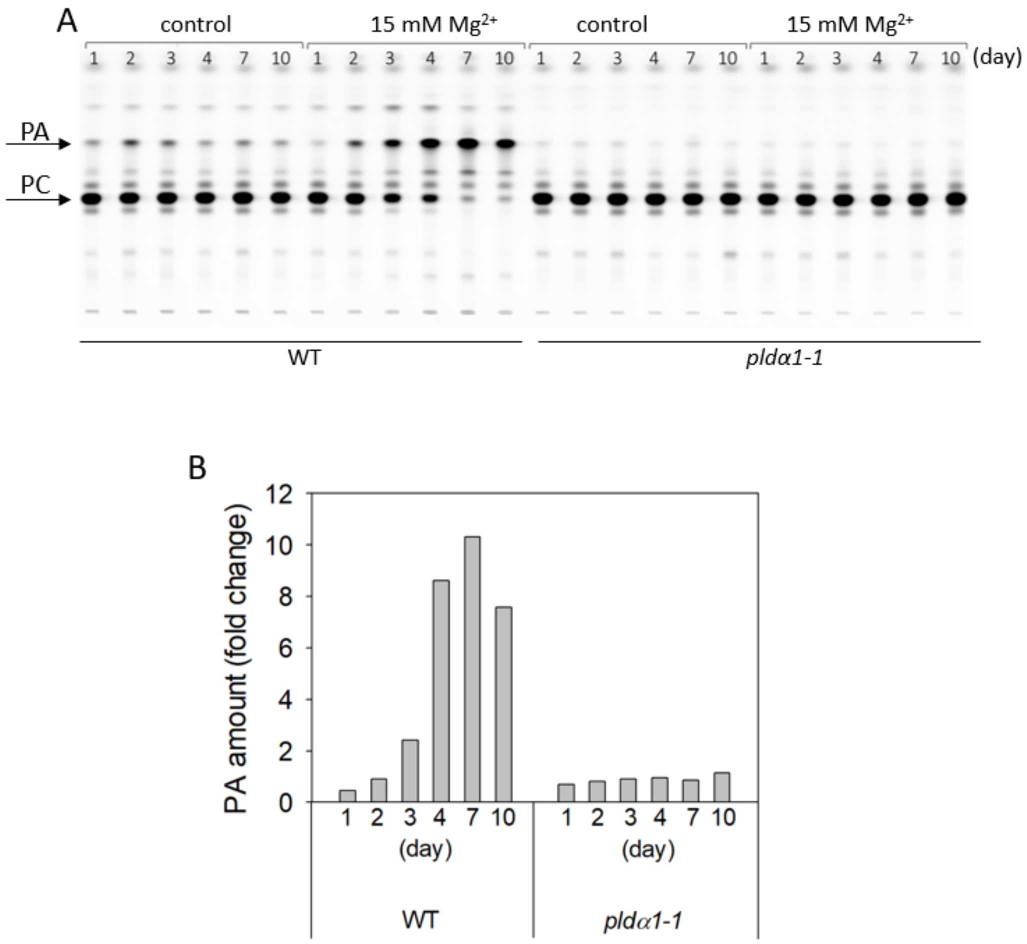

Figure S1. PA amount increases in response to high-Mg^{2+} stress in wt but not in *pldα1-1*. 24-day-old hydroponically grown plants were treated with 15 mM $MgSO_4$ and sampled after 1, 2, 3, 4, 7 and 10 days after the treatment. (A) Thin layer plate showing phosphatidic acid, product of PLDα1 activity. PLDα1 activity was measured in leave extracts from leaves 3-6 from plants treated with $MgSO_4$. (B) Relative amount of PA with $MgSO_4$ treatment over time. PA, phosphatidic acid, PC, phosphatidylcholine.

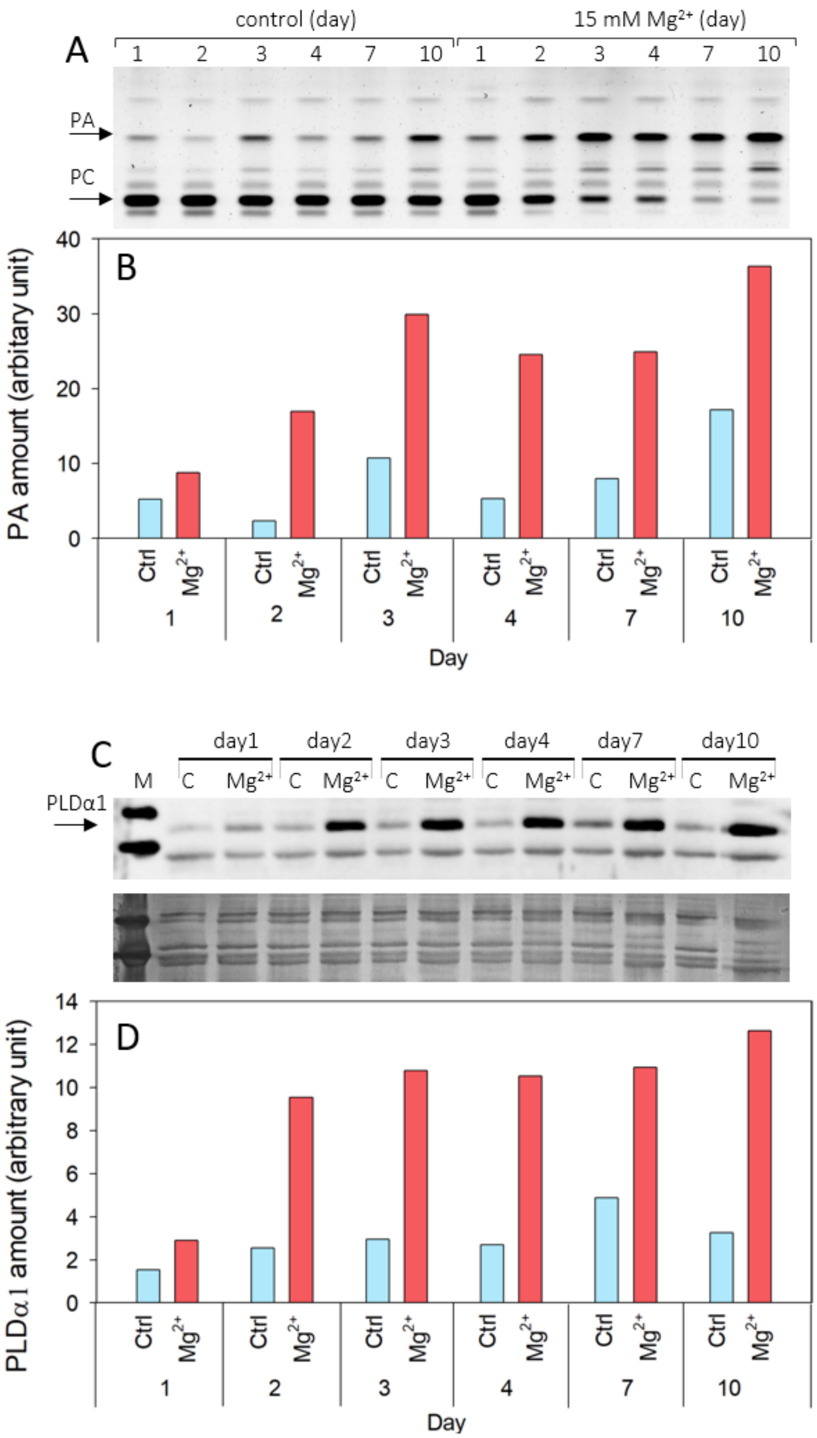

Figure S2. Phospholipase Dα1 amount and activity increases in response to high Mg^{2+} stress in youngest leaves. 24-day-old hydroponically grown plants were treated with 15 mM $MgSO_4$ and sampled after 1, 2, 3, 4, 7 and 10 days after the treatment. (A) Thin layer plate showing phosphatidic acid, product of PLDα1 activity. PLDα1 activity was measured in leave extracts from leaves 7-10 from plants treated with $MgSO_4$. (B) Increase of PA with $MgSO_4$ treatment over time. (C) Western blot detection of PLDα1 in protein extracts from leaves. Each lane was run with 10 µg of protein, upper panel – western blot, lower panel – membrane stained with Novex reversible membrane protein stain, C-control. (D) Quantification of PLDα1 protein. The experiments were repeated 3 times with similar results. PA, phosphatidic acid, PC, phosphatidylcholine, M, molecular marker.

Table S1: List of primers

List of primers used for quantitative RT-PCR			
Gene	Locus	Forward primer	Reverse primer
ANAC092[1]	At5g39610	cttaccatggaaggctaagatggg	ttccaataaccggcttctgtcg
BAM1[2]	At3g23920	gatcacgaacagcctcaaga	gagaatctgctcgtgtgcat
BAM2[2]	At4g00490	ggagctccgacgagagtatc	gagaaccaaaggcctagcac
CBL1[3]	At4g17615	cctctgagacagcttttagtgtg	tatattctccctcttccggcttt
CIPK23[2]	At1g30270	ggaaaaccatcgacgaagag	ggttagccatggcaccatta
SAG13[1]	At2g29350	agggagcatcgtgctcatatcc	ccagctgattcatggctcctttg
SAND family protein[4]	At2g28390	ggattttcagctactcttcaagcta	ctgccttgactaagttgacacg

1 **Bresson J, Bieker S, Riester L, Doll J, Zentgraf U.** 2018. A guideline for leaf senescence analyses: from quantification to physiological and molecular investigations. Journal of Experimental Botany **12**, 769-786.

2 this study

3 **Mähs A, Steinhorst L, Han J-P, Shen L-K, Wang Y, Kudla J.** 2013. The calcineurin B-Like Ca^{2+} sensors CBL1 and CBL9 function in pollen germination and pollen tube growth in *Arabidopsis*. Molecular Plant **6**, 1149-1162.

4 **Ginglinger J.-F., Boachon B., Höfer R., Paetz C., Köllner T.G., Miesch L., Lugan R., Baltenweck R., Mutterer J., Ullmann P., Beran F., Claudel P., Verstappen F., Fischer M.J.C., Karst F., Bouwmeester H., Miesch M., Schneider B., Gershenzon J., Ehlting J., Werck-Reichhart D.** 2013. Gene coexpression analysis reveals complex metabolism of the monoterpene alcohol linalool in *Arabidopsis* flowers. Plant Cell **25**, 4640-4657.
